# Evaluation of biochemical markers during somatic embryogenesis in *Silybum marianum* L.

**DOI:** 10.1007/s13205-016-0366-1

**Published:** 2016-02-16

**Authors:** Bilal Haider Abbasi, Huma Ali, Buhara Yücesan, Sabahat Saeed, Khalid Rehman, Mubarak Ali Khan

**Affiliations:** 1 Department of Biotechnology, Quaid-i-Azam University, Islamabad, 45320 Pakistan; 2 Department of Biotechnology, Bacha Khan University, Charsada, KP Pakistan; 3 Faculty of Natural and Agricultural Sciences, Department of Seed Science and Technology, Abant Izzet Baysal University, 14030 Bolu, Turkey; 4 Biotechnology Program, Department of Environmental Sciences, COMSATS Institute of Information Technology (CIIT), Abbottabad, Pakistan; 5 Department of Plant Sciences, Quaid-i-Azam University, Islamabad, 45320 Pakistan

**Keywords:** Silybum, Explant, Somatic embryo, Plant growth regulator, Biochemical, HPLC

## Abstract

In present report effects of explants type, basal media and plant growth regulators (PGRs) were tested for induction of indirect somatic embryogenesis in medicinally important plant *Silybum marianum* L. Leaf, petiole and root explants were exploited in vitro on B5 (Gamborg), SH (Schenk and Hildebrandt) and MS (Murashige and Skoog) media for induction of embryogenic callus followed by somatic embryogenesis. Highest callus induction frequency (76 ± 4.8 %) was recorded when petiole explants of in vitro derived plantlets were cultured on B5 medium supplemented with 1.5 mg l^−1^ 2,4-dichlorophenoxyacetic acid (2,4_D) in combination with 1.5 mg l^−1^ Thidiazuron (TDZ). Induction and multiplication of somatic embryos were observed, when the embryogenic calluses were sub-cultured on to B5 medium containing 0.5 mg l^−1^ 2,4-D plus 1.5 mg l^−1^ TDZ. At this PGRs treatment, 77 % of the cultures responded with 39.1 somatic embryos per callus. Furthermore, MS0 medium was indicated more reponsive for growth and maturation of somatic embryos. Analysis of biochemical markers during various growth phases in somatic embryogenesis revealed that somatic embryos exhibited highest level of total carbohydrate, starch, ascorbic acid and total free amino acids. However, higher protein levels were detected in non-embryogenic callus. Nevertheless, considerable amount of silymarin (4.1 mg g^−1^ DW) was detected in somatic embryos than other growth phases. Thus, the present study concluded that biochemical and physiological changes during embryogenesis are influenced by interplay of explants type, basal media and PGRs.

## Introduction

*Silybum marianum* (L.) Gaertn. (Milk thistle) of Asteracea family is valued for its bioactive compound called silymarin and has been used globally for the treatment of hepatic disorders for centuries (Khan et al. 2013). Silymarin consists of the isomers of different flavanolignans, having health promoting activities such as anti-oxidant, anti-inflammatory, anti-hepatitis, anti-bacterial and anti-viral (Abbasi et al. 2010). The exhibiting higher variability in the phytochemical contents in wild grown medicinal plants is the major bottleneck in pharmaceutical preparations for production of effective herbal medicines (Khan et al. 2015a). Similarly, the wild grown *Silybum* plants possess the problems of low efficacy in silymarin due to geographic variability, contamination, herbal adulteration and lack of uniform procedures for cultivation and sustainable harvest (Haban et al. 2009). However, development of elite varieties with predictable phytochemical profiles towards plant tissue culture application might probably circumvent these issues of variability in *Silybum* end products (Khan et al. 2014). Clonal plant production in vitro has potential to ensure vigorous growth of pharmacologically superior plants, and thus significantly reduce contamination of plants, and facilitate biochemical characterization supported by chromatographic fingerprint analysis for quality control (Murch et al. 2006). The biotechnological interventions for establishment of a feasible system of somatic embryogenesis may produce uniform plants rapidly and easily (Moon et al. 2013). The process of differentiation is a consequence of some biochemical and physiological changes induced by plant growth regulators; therefore evaluation of biochemical parameters in different growth phases during somatic embryogenesis could be used as markers for monitoring different events taking place during the process of somatic embryogenesis (Jeyaseelan and Rao 2005).

The present study aimed to establish a feasible method for somatic embryogenesis using different explants, basal media containing various combinations and concentrations of PGRs; furthermore, to evaluate the levels of carbohydrates, proteins, amino acids, phenolic compounds and silymarin content in different growth phases during somatic embryogenesis of *S. marianum*.

## Materials and methods

### Formation of embryogenic callus

Mature and viable seeds of *S. marianum* were in vitro germinated as per the reported protocol of Khan et al. (2013). In preliminary studies three different culture media were tested for callus induction including MS (Murashige and Skoog 1962), SH (Schenk and Hildebrandt 1972) and B5 medium (Gamborg et al. 1968). Due to high frequency of callus formation in all types of explants, B5 medium was selected for subsequent experiments accordingly. Leaf explants (~1.5 cm^2^), petiole explants (~2.0 cm) and root segments (~0.5 cm) were taken from 4-week-old in vitro germinated seedlings, and then placed onto B5 media containing 3 % sucrose (w/v) and 0.8 % (w/v) agar in 150 ml conical flask supplemented with [0.5, 1.0, 1.5, 2.0 or 2.5 mg l^−1^] of 2,4-D or TDZ alone or 1.5 mg l^−1^ TDZ in combination with 2,4-D [0.5, 1.0, 1.5, 2.0 or 2.5 mg l^−1^]. The pH of media was adjusted to 5.8 prior to autoclaving (121 °C, 20 min at 1 atm. pressure), cultures were placed in 16 h photoperiod with light intensity of ~40 μmol m^−2^ s^−1^ and temperature was maintained at 25 ± 1 °C. In all sets of experiments, PGR-free medium was used as control treatment during callus induction. After 4 weeks of callus induction, the frequency of callus induction (%) was recorded.

### Induction and maturation of somatic embryos

The embryogenic calli were aseptically cut into small sections (2 cm) and were transferred into B5 medium containing [0.5, 1.0 or 1.5 mg l^−1^] 2,4-D alone or in combination with [1.5, 1.0 or 0.5 mg l^−1^] TDZ. To investigate the impact of type of media on the maturation of somatic embryos, another set of basal media [MS0 or ½ MS (Murashige and Skoog 1962)] without PGRs was used. The percent maturation of somatic embryos and their growth were recorded after 4 weeks of culture in a flask with three replications.

### Biochemical assays

Plant samples raised in vitro were denoted on the basis of the growth patterns during embryogenesis as non-embryonic callus (NEC), Embryogenic callus (EC) and Somatic embryos (SE). Fresh plant tissues were used for the extract preparation as per the valuable protocol of Jeyaseelan and Rao (2005). Briefly, 1 g of fresh plant material of each sample was homogenized in 2 ml of 50 mM potassium phosphate buffer, pH 7.5, containing 2 mM EDTA in pestle and mortar. The extract was centrifuged at 15,000 rpm for 20 min at 4 °C and supernatant was collected and used for analysis of biochemical markers. UV–visible spectrophotometer (Halo DR-20, UV–Vis spectrophotometer, Dynamica Ltd, Victoria, Australia) was used to determine absorption of extracts. Total carbohydrate and soluble sugars were estimated by the method of Dubois et al. (1956), Starch content was determined by the method of Cready (1950), Protein levels were estimated by the method of Lowry et al. (1951), free amino acids were determined by the method of Yemm et al. (1955), proline and glutamine were determined by the method of Bates et al. (1973), ammonia was estimated by the method Okamura et al. (1975), phenols were determined by the method of Swain and Hillis (1959) and ascorbic acid content was estimated by the method of Gillespie and Ainsworth (2007).

### Silymarin extraction and HPLC analysis

Analysis of silymarin content in the in vitro raised plant samples was carried out according to the method of Khan et al. (2013). Finely ground and dried plant material (200 mg) of each sample was ultrasonicated in solvent mixture contained methanol (CH_4_O) and 0.1 % phosphoric acid (H_3_PO_4_) in a ratio of 70 V:30 V, (1 mil each) for 30 min. We used Shimadzu Lc8A system for HPLC (High Performance Liquid Chromatography) set up with a binary pump, solvent vacuum degasser, a variable wavelength (*λ*) detector, and an auto sampler containing an injection loop (10 μl). The column used was C18 (ODS) with particle size (150 × 4.6 mm) and the chromatographic eluents consisted of ultrapure water containing 0.1 % H_3_PO_4_ (Pump A) and C_2_H_3_N; acetonitrile (pump B). The scheme for gradient elution of silymarin was set as: 0–30 min, 10–20 % B; 30–110 min, 20–80 % B. The rate of elution flow was kept as 1.0 ml/min with the injection volume of 10 µl. Silymarin (Sigma; CA, USA) was used as a standard reference and samples were analyzed on the basis of comparison of peak areas and retention times of the samples with that of the standard. The content of silymarin was quantified and expressed in mg g^−1^ DW (dry weight).

### Experimental design and statistical analysis

All experiments were repeated twice and each treatment consisted of three replicates. Mean values of various treatments were subjected to analysis of variance (ANOVA). Statistix software (8.1 versions; USA) was used to calculate standard errors (±) and least significant difference (LSD) while Origin Lab (8.5) software was used for graphical presentation.

## Results and discussion

### Influence of type of explants and basal media on induction of embryogenic callus

**Fig. 1:**
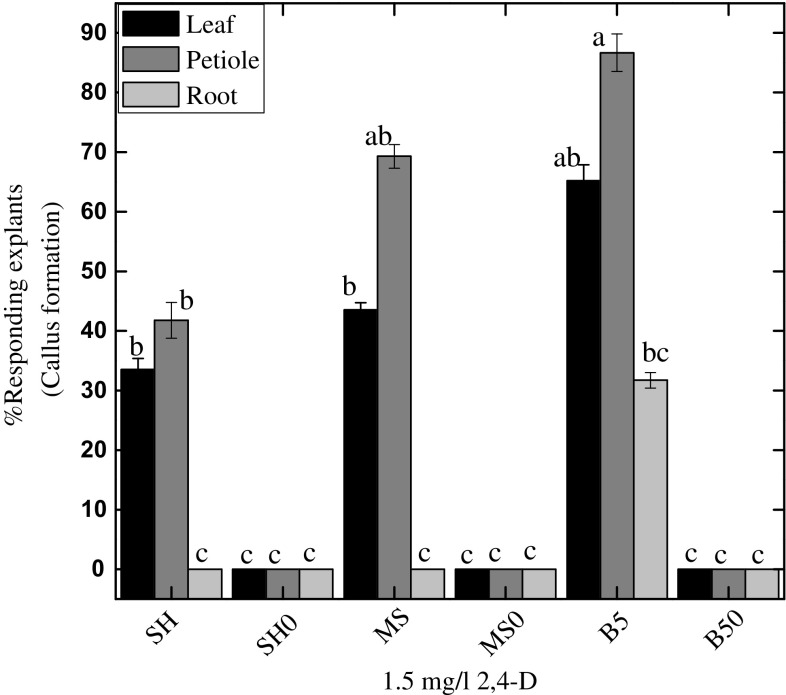
Effects of various growth media with or without 1.5 mg l^−1^ 2,4-D on explants. Data were collected after 4 weeks of culture. Values are the mean ± standard error from three replicates. *Column bars* sharing the *same English letter/s* are similar otherwise differ significantly at *P* < 0.05

Callus formation was induced from all explant types tested on B5 medium while MS or SH was only effective in leaf and petiole explants (Fig. 1). Maximum callus formation frequency (88 %) was observed for petiole explants followed by leaf explants (68 %); however, root explants failed to induce callus on both MS and SH media augmented with 1.5 mg l^−1^ 2,4-D. No callus formation was observed in control treatments for all the media and explants tested (Fig. 1). Due to its highest efficacy in callus formation, B5 medium was selected for subsequent experiments. The differential response by different media in our study can be anticipated to the different salt content contained in these media. Medium types can significantly influence on the efficiency of callus induction (Rodriguez-Sahagun et al. 2011). Hence, selection of suitable media is a pre-requisite step for establishment of a feasible protocol of somatic embryogenesis in plants (Pinto et al. 2008).

### Influence of type of explants and PGRs on induction of embryogenic callus

Highest callus formation frequency (86.7 %) was observed when petiole explants were incubated on B5 medium containing 1.5 mg l^−1^ 2,4-D. Callus formation was started at the cut ends of petiole segments after 1 week of culture cultivation (Table 1). 2,4-D has also proven a potent bio-regulator for induction of embryogenic callus in *Leucojum aestivum* (Ptak et al. 2013). Supplementation of culture flasks with 1.5 mg l^−1^ 2,4-D in combination with 1.5 mg l^−1^ TDZ significantly enhanced the callus formation in petiole explants. However, leaf explants were also susceptible to callus formation by the application of 1.5 mg l^−1^ 2,4-D either alone or in combination with 1.5 mg l^−1^ TDZ. Less callus formation (29 %) was detected in root explants (Table 1). Formation of somatic embryos was not observed on any of the induced callus. The calli were sub-cultured on the meida with similar hormonal treatment for induction of embryogenic callus. Sub-culturing of the calli derived from petiole explants on B5 medium containing 1.5 mg l^−1^ 2,4-D plus 1.5 mg l^−1^ TDZ favored highest embryogenic potential (77 %; Table 1). However calli derived from leaf explants showed moderate embryogenic potential (51 %) when incubated at the aforementioned PGRs treatment (Table 1). After 2-week culture period, the callus explants were surrounded by proliferating embryogenic callus. However, the calli induced from root explants failed to produce embryogenic calli (Table 1). These differences in response of explants to the application of various PGRs in our study might be related to the variability in genetic makeup of the explants (Sakhanokho et al. 2005). Furthermore, the interaction of explants with plant growth regulators in culture medium may decide the success of plant regeneration through indirect somatic embryogenesis, supporting the significance of selection of suitable explant type (Moon et al. 2013).

**Table 1:** Effects of various concentrations and combinations of 2,4-D and TDZ in B5 media on embryogenic callus formation from leaf, petiole and root explants

PGRs (mg g^−1^)	Callus Induction frequency (%)			Embryogenic callus formation (%)	
	Leaf	Petiole	Root	Leaf	Petiole
0.5 2,4-D	28.3 ± 1.5	69.6 ± 4.6	15.7 ± 0.9	18 ± 2.4	18.2 ± 1.2
1.0 2,4-D	47.4 ± 4.2	73.4 ± 5.4	22 ± 1.9	28 ± 1.5	28.4 ± 2.6
1.5 2,4-D	65.2 ± 5.0	86.2 ± 6.5	31 ± 2.0	45.2 ± 3.1	46 ± 3.2
2.0 2,4-D	44.7 ± 3.1	75.1 ± 5.9	28 ± 1.5	36 ± 2.9	31 ± 1.4
2.5 2,4-D	38.2 ± 2.1	67.2 ± 4.3	19.2 ± 2.5	23 ± 2.4	21.1 ± 0.8
0.5 TDZ	18.2 ± 2.4	22.1 ± 1.0		14.4 ± 0.2	28 ± 1.5
1.0 TDZ	25.3 ± 1.9	39 ± 2.2		19.3 ± 2.5	36 ± 1.2
1.5 TDZ	34.2 ± 2.1	59.4 ± 5.3		28 ± 1.5	49 ± 1.8
2.0 TDZ	29.1 ± 1.6	46 ± 4.0		22 ± 2.0	22.7 ± 1.3
2.5 TDZ	18.2 ± 2.4	22.2 ± 1.0		16.1 ± 0.8	16.3 ± 0.8
0.5 2,4-D + 2.5 TDZ	46.4 ± 4.0	63 ± 4.5	26 ± 2.0	36 ± 3.0	43 ± 3.7
1.0 2,4-D + 2.0 TDZ	62 ± 4.9	76.1 ± 5.7	34.3 ± 2.1	51 ± 4.2	58.1 ± 2.2
1.5 2,4-D + 1.5 TDZ	76 ± 4.8	90 ± 7.6	29 ± 1.6	39.3 ± 2.2	77 ± 6.2
2.0 2,4-D + 1.0 TDZ	42.2 ± 4.2	78.4 ± 5.1	21.1 ± 1.3	31 ± 2.0	52.1 ± 3.4
2.5 2,4-D + 0.5 TDZ	39.3 ± 2.2	64 ± 4.4	14.4 ± 0.2	22.6 ± 1.9	39.2 ± 2.6

Data on embryogenic callus formation were recorded after 2 weeks of culture when 4 week old calli were sub-cultured on same media. Values are the mean ± standard error from three replicates

### Induction and maturation of somatic embryos

**Fig. 2:**
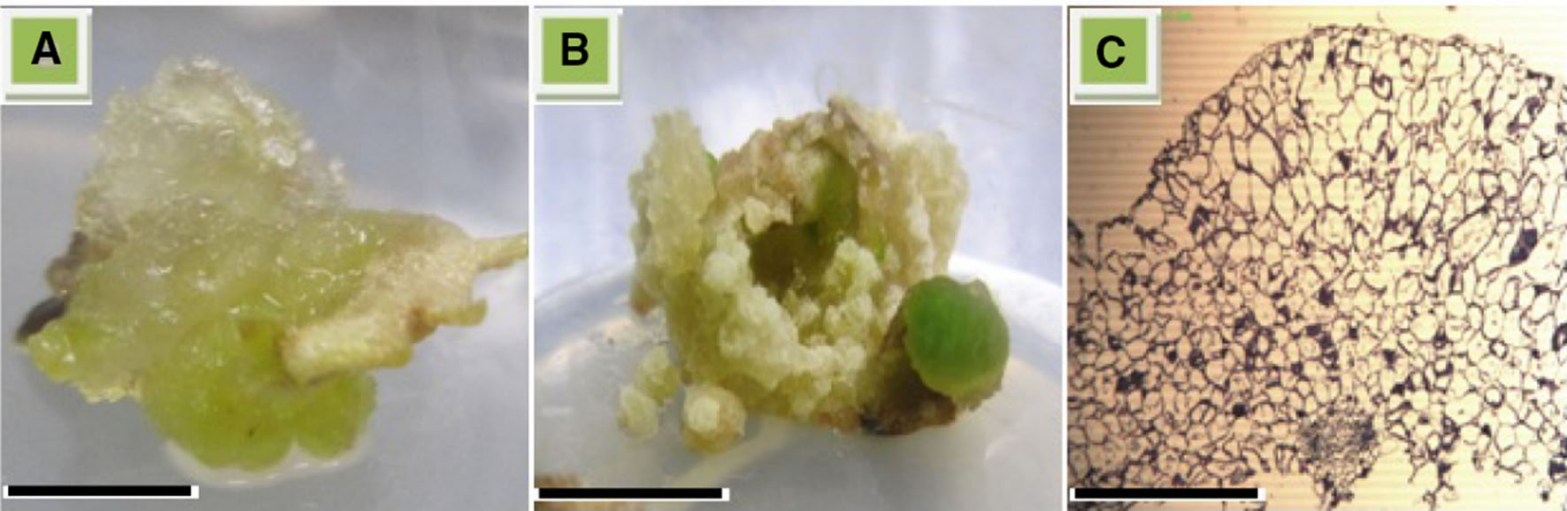
Somatic embryogenesis in *Silybum marianum*. **a** Callus formation at cut ends of petiole explants after 1 week of culture (*bar* = 2 mm). **b** Embryogenic callus after 2 weeks of culture period (*bar* = 2 mm). **c** Induction of somatic embryogenesis,embryonic cells showing isodiametric cells while non-embryonic cells have large vacuole, small starch granules and abundant intercellular spaces (*bar* = 250 µm)

**Fig. 3:**
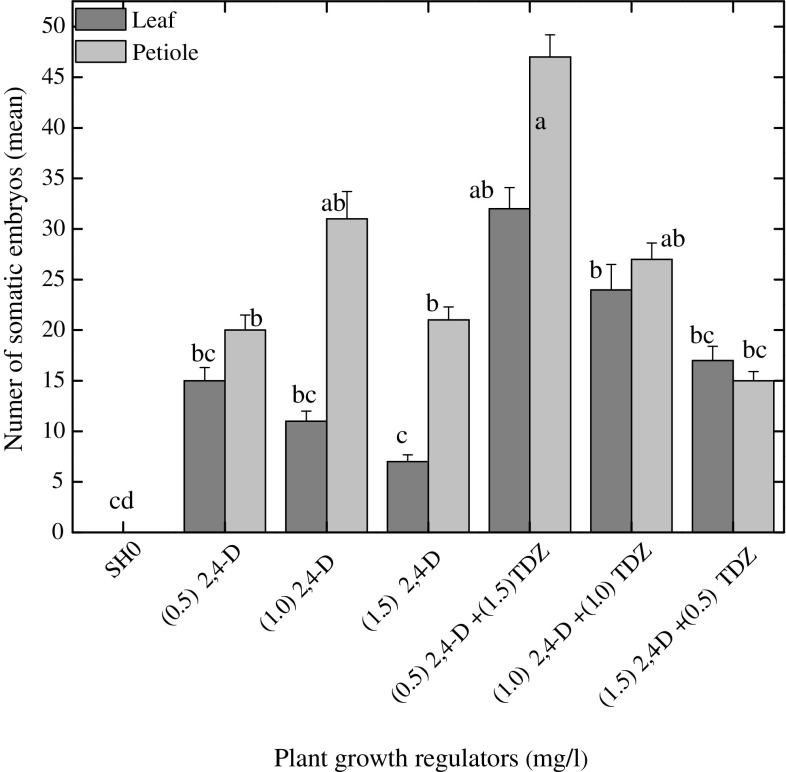
Effects of various concentrations and combinations of 2,4-D and TDZ in B5 media on mean number of somatic embryos per embryogenic callus from leaf and petiole explants. Data were collected after 4 weeks of culture. Values are the mean ± standard error from three replicates. *Column bars* sharing the *same English letter/s* are similar otherwise differ significantly at *P* < 0.05

**Fig. 4:**
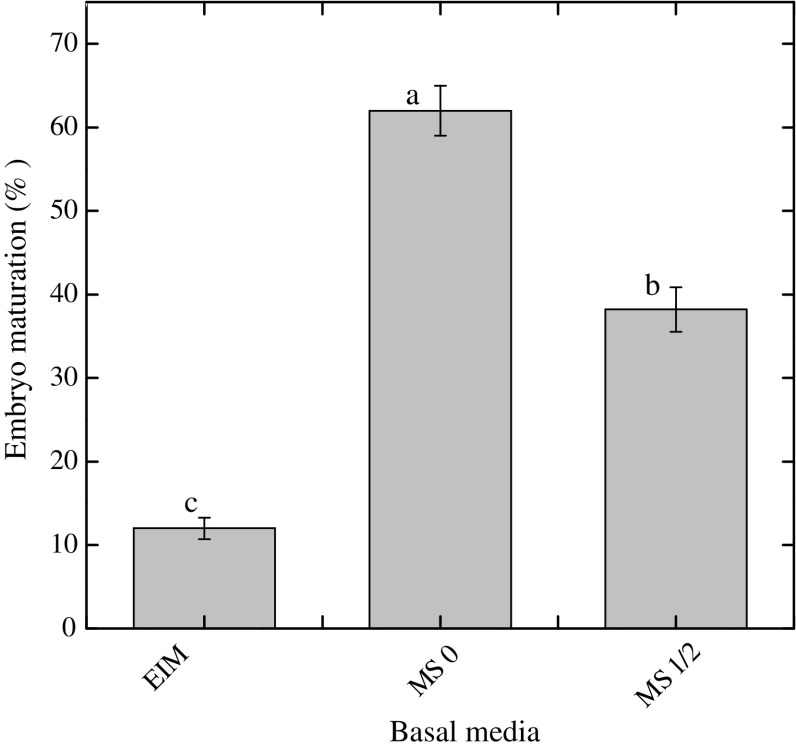
Effects of various growth media on somatic embryo maturation (%). Data were collected after 2 weeks of culture. *EIM* Embryo induction medium. Values are the mean ± standard error from three replicates. *Column bars* sharing the *same English letter/s* are similar otherwise differ significantly at *P* < 0.05

Maximum numbers of somatic embryos (31 somatic embryo per callus) were inspected at 2,4-D alone (1.0 mg l^−1^) from petiole derived embryogenic callus (Fig. 3). Similarly, 2,4-D has been reported as the most effective auxin for induction of somatic embryos in many medicinal plants (Prange et al. 2010; Sivanesan et al. 2011; Zhang et al. 2010). The somatic embryos were observed on the outermost cell layer of the callus within 2–3 weeks after transfer into embryo induction medium (Fig. 2b). The number of somatic embryos per callus was further enhanced, when 2,4-D was combined with TDZ in both petiole and leaf explants (Fig. 3). Embryo maturation is an important phase in development of somatic embryo and is influenced by various morphological and biochemical changes in embryo-like deposition of storage materials, size enlargement and acquaintance of germination (Moon et al. 2013). The embryogenic calli with developed somatic embryos were cut aseptically into small sections (~2.5 cm^2^) and were subsequently transferred into fresh medium of same composition. No further growth in somatic embryos was observed when cultured on same medium (Fig. 4). An adequate change in response to embryo maturation (62 %) was observed when the embryos were transferred from embryo induction medium into MS0 medium (Fig. 4). It can be extrapolated from our study that once embryogenesis starts, embryo begins to synthesize its own auxins and thus require less or no auxin. Sometimes the maturation and germination of somatic embryos into plantlets are difficult to attain during embryogenesis, such problems can act as the major barriers in the application of somatic embryogenesis systems for commercial purposes (Moon et al. 2006).

### Analysis of biochemical markers

Significant variations in biochemical parameters were observed during somatic embryogenesis, which could be used as markers for monitoring the different events taking place during the process of somatic embryogenesis.

#### Levels of carbohydrates

The highest level of total carbohydrate (1620 μg/g FW) was detected in SE, and lowest value was detected in NEC (Table 2). However, the level of total soluble sugar (1205 μg/g FW) was maximum in NEC with a descending order in activity from NEC to SE. Decrease in sugar content might be associated with utilization of sugars in different growth phases during somatic embryogenesis (Correia et al. 2012).

**Table 2:** Biochemical markers (µg/g FW) in in vitro-grown plant samples collected from different growth phases during somatic embryogenesis in *Silybum marianum*

Biochemical markers (µg g^1^ fw)	NEC	EC	SE
Total carbohydrate	514 ± 38.5	1100 ± 65.2	**1620** **±** **99.3**
Total soluble sugars	**1205** **±** **78.9**	878 ± 33.8	613 ± 24.4
Starch	378 ± 22.9	685 ± 49.2	**1035** **±** **76.6**
Proteins	**444** **±** **74.3**	303 ± 29.4	151 ± 22.3
Total free amino acids	132 ± 18.5	283 ± 25.6	**412** **±** **38.3**
Proline	137 ± 18.4	**550** **±** **57.8**	385 ± 43.6
Glutamine	48 ± 7.3	95 ± 16.7	**112** **±** **19.8**
Ammonia	**88** **±** **21.6**	57 ± 14.5	41 ± 15.1
Phenols	6 ± 0.8	**22** **±** **2.8**	16 ± 1.7
Ascorbic acid	8 ± 1.2	17 ± 0.9	**28** **±** **1.9**

Values are the mean ± standard error from three replicates

Bold values represent the highest amount of each biochemical marker

*NEC* Non-embryogenic callus, *EC* Embryogenic callus, *SE* Somatic embryo

An exponential increase in starch level from non-embryogenic callus (NEC) to embryogenic callus (EC) and into somatic embryos (SE) was observed in current study (Table 2). The enhanced accumulation of starch in somatic embryos is probably the result of a modification in cellular metabolism that might be a consequence of a high sucrose levels used in the culture medium (Pinto et al. 2011). Similarly, accumulation of sugars and starch were higher in embryogenic callus than those of non-embryogenic callus (Naidu and Kishor 1995).

#### Nitrogenous compounds

Higher total protein levels (444 μg/g FW) were found in NEC (Table 2). In the process of embryogenesis, the total protein content was significantly higher in non-differentiating callus. The gradual decline in the protein content during embryogenic phases clearly shows the utilization of high protein content at the stage of embryo induction. Highest protein content in NEC has also been reported in *cumin* plant somatic embryogenesis by Dave and Batra (1995). Decreased protein levels were also reported in somatic embryos of soybean (Chanprame et al. 1998). Data presented in (Table 2) shows that somatic embryos secreted highest levels of total free amino acids (412 μg/g FW), however, proline level was maximum in EC (550 μg/g FW) and glutamine level (112 μg/g FW) was significantly higher in SE. Glutamine is an important amino acid involved in various biosynthetic pathways for plant metabolism (Khan et al. 2015b). In our study, elevating level of ammonia was detected in NEC. Previously, tryptophan, proline and serine along with ammonium ion were reported to foster the development of somatic embryos in diverse taxa like *Medicago sativa* (Stuart et al. 1985).

#### Phenolics and ascorbic acid

Phenolic content was higher (22 μg/g FW) in EC while increased level of ascorbic acid content (28 μg/g FW) was detected in SE when compared to other growth lines (Table 2). Ascorbic acid is a strong antioxidant, controls the color pigmentation of the embryogenic callus and is involved in plant antioxidant system to cope any stress condition (Khan et al. 2015b). Additionally ascorbic acid has long been recognized as a strong antioxidant for its role in oxidative phosphorylation and photophosphorylation, stimulation of RNA synthesis, bud development and prevention of senescence (Rathod et al. 2012).

### Silymarin content

**Fig. 5:**
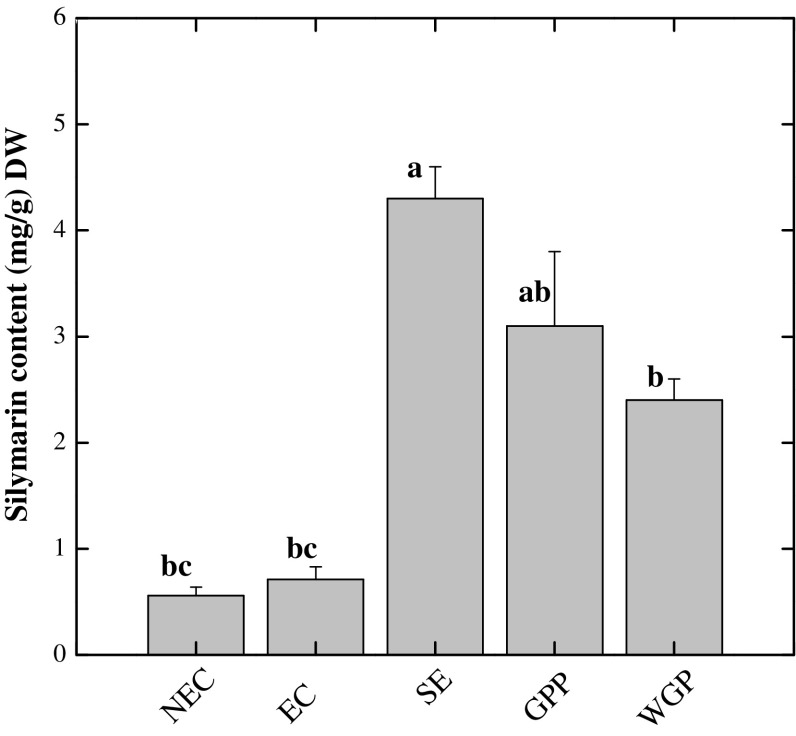
Silymarin content (mg g^−1^ DW) in in vitro-grown plant samples collected from different growth phases during somatic embryogenesis in *Silybum marianum*. Values are the mean ± standard error from three replicates. *Column bars* sharing the *same English letter/s* are similar otherwise differ significantly at *P* < 0.05

Highest content of silymarin (4.1 mg g^−1^ DW) was detected in SE. However, there was no significant difference (*P* < 0.05) in silymarin content between growth room potted plantlets (GPP) and wild grown plants (WGP) (Fig. 5). Moreover our data demonstrates that differentiated tissues exhibited higher level of silymarin than non differentiating tissues. Nevertheless, cell cultures of *S. marianum* are more competent for biosynthesis of silymarin in vitro, but in lesser amounts than wild grown plants (Abbasi et al. 2010). Numerous reports indicated higher accumulation of silymarin in the regenerated plant tissues in comparison to wild grown plants. (Khan et al. 2013, 2014; El Sherif et al. 2013). As wild grown *silybum* plants are susceptible to seasonal variations and environmental pollutants which may affect the medicinal efficiency of the harvested tissues, however the in vitro production of plant secondary metabolites under controlled conditions can be a good strategy for formulation of effective herbal medicines (Khan et al. 2013). The concentrations of various secondary plant products strongly depend on the growth conditions and it is obvious that in vitro stress conditions have a strong impact on the metabolic pathways responsible for the accumulation of the related natural products (Khan et al. 2015b). In corroboration to data of biochemical markers, it is evidenced that silymarin production in SE was synchronized by the profound expression of other phytochemicals like sugars, starch and ascorbic acid (Table 2). Our data is comparative to Khan et al. (2013), they have reported 5.48 mg g^−1^ DW silymarin in in vitro germinated plantlets. Moreover, El Sherif et al. (2013) observed considerable amount of silymarin content in multiple shoot cultures of *S. marianum* by different biotic elicitors. Since silymarin biosynthesis involves the conversion of phenylalanine to cinnamic acid by phenyl ammonia lyase (PAL) enzyme (Khan et al. 2015b). Therefore it might be hypothesized that PAL enzyme has been triggered during SE growth phase for signaling the phenylpropanaoid pathway to circumvent the in vitro oxidative stress by production of higher level of silymarin for development of normal somatic embryos during embryogenesis.

## Conclusions

This paper reports an efficient procedure for somatic embryogenesis in *S. marianum*. Indirect somatic embryogenesis through embryogenic callus formation was observed. Embryogenic callus formation and embryo induction and multiplication were achieved on B5 media while embryo maturation was achieved on MS zero medium. Significant variations were detected in biochemical studies in plant samples raised from different growth phases. Moreover, considerable content of silymarin (4.1 mg/g DW) was detected in somatic embryos by HPLC. Future research shall refine this technique for achieving higher frequencies of embryo proliferation to allow long term culture maintenance.

## Abbreviations

SH: Schenk and Hildebrandt
MS: Murashige and Skoog
PGR: Plant growth regulator
SE: Somatic embryo
NEC: Non-embryogenic callus
FW: Fresh weight
DW: Dry weight
